# Association between SARS-CoV-2 gene specific Ct values and COVID-19 associated in-hospital mortality

**DOI:** 10.3389/fepid.2024.1375975

**Published:** 2024-04-26

**Authors:** Mpho L. Sikhosana, Richard Welch, Alfred Musekiwa, Zinhle Makatini, Joy Ebonwu, Lucille Blumberg, Waasila Jassat

**Affiliations:** ^1^ Department of Virology, National Health Laboratory Service, Charlotte Maxeke Johannesburg Academic Hospital, Johannesburg, South Africa; ^2^ Department of Virology, Faculty of Health Sciences, University of the Witwatersrand, Johannesburg, South Africa; ^3^ Department of Public Health and Outbreak Response, National Institute for Communicable Diseases, Johannesburg, South Africa; ^4^ Faculty of Health Sciences, School of Health Systems and Public Health, University of Pretoria, Pretoria, South Africa

**Keywords:** SARS-CoV-2, Ct values, mortality, South Africa, PCR

## Abstract

**Background:**

Since there are currently no specific SARS-CoV-2 prognostic viral biomarkers for predicting disease severity, there has been interest in using SARS-CoV-2 polymerase chain reaction (PCR) cycle-threshold (Ct) values to predict disease progression.

**Objective:**

This study assessed the association between in-hospital mortality of hospitalized COVID-19 cases and Ct-values of gene targets specific to SARS-CoV-2.

**Methods:**

Clinical data of hospitalized COVID-19 cases from Gauteng Province from April 2020-July 2022 were obtained from a national surveillance system and linked to laboratory data. The study period was divided into pandemic waves: Asp614Gly/wave1 (7 June–22 Aug 2020); beta/wave2 (15 Nov 2020–6 Feb 2021); delta/wave3 (9 May–18 Sept 2021) and omicron/wave4 (21 Nov 2021–22 Jan 2022). Ct-value data of genes specific to SARS-CoV-2 according to testing platforms (Roche-ORF gene; GeneXpert-N2 gene; Abbott-RdRp gene) were categorized as low (Ct < 20), mid (Ct20–30) or high (Ct > 30).

**Results:**

There were 1205 recorded cases: 836(69.4%; wave1), 122(10.1%;wave2) 21(1.7%; wave3) and 11(0.9%;in wave4). The cases' mean age(±SD) was 49 years(±18), and 662(54.9%) were female. There were 296(24.6%) deaths recorded: 241(81.4%;wave1), 27 (9.1%;wave2), 6 (2%;wave3), and 2 (0.7%;wave4) (*p* < 0.001). Sample distribution by testing platforms was: Roche 1,033 (85.7%), GeneXpert 169 (14%) and Abbott 3 (0.3%). The median (IQR) Ct-values according to testing platform were: Roche 26 (22–30), GeneXpert 38 (36–40) and Abbott 21 (16–24). After adjusting for sex, age and presence of a comorbidity, the odds of COVID-19 associated death were high amongst patients with Ct values 20–30[adjusted Odds Ratio (aOR) 2.25; 95% CI: 1.60–3.18] and highest amongst cases with Ct-values <20 (aOR 3.18; 95% CI: 1.92–5.27), compared to cases with Ct-values >30.

**Conclusion:**

Although odds of COVID19-related death were high amongst cases with Ct-values <30, Ct values were not comparable across different testing platforms, thus precluding the comparison of SARS-CoV-2 Ct-value results.

## Introduction

Since the COVID-19 pandemic emerged, the diagnosis of SARS-CoV-2 has primarily been based on the detection of SARS-CoV-2 RNA gene sequences using reverse transcription polymerase chain reaction (rRT-PCR) testing (1). rRT-PCR is a molecular diagnostic test that amplifies the virus' nucleic acid through the process of thermocycling (2). The number of amplification cycles required for the fluorescence of a gene target to exceed a pre-determined detection threshold level is called the cycle threshold (Ct) value of that particular gene target. The Ct value correlates inversely to the viral load, such that lower Ct values are associated with higher viral loads (2, 3). At the time that this paper was written, there was no Food and Drug Administration (FDA) approved quantitative assay used for reporting absolute SARS-CoV-2 viral copy numbers (4, 5), nor were there any SARS-CoV-2-specific prognostic viral biomarkers available for predicting severe outcomes amongst cases (6). However, evidence from previous studies have shown that higher initial viral loads of SARS-CoV-1 and influenza have been associated with worsening disease course (6), and this could presumably also apply to SARS-CoV-2.

Although the impact of SARS-CoV-2 rRT-PCR test cycle threshold (Ct) values on adverse outcomes following COVID-19 infection has been investigated, there is currently no consensus regarding the clinical utility of SARS-CoV-2 Ct values. Our study therefore aimed to determine whether there is an association between Ct values of gene targets specific to SARS-CoV-2 and in-hospital COVID-19 related mortality.

## Methods

This was a sub-study of a parent protocol titled “DATCOV: A surveillance programme for hospitalised and Care Home individuals with COVID-19 in South Africa 2020”. We conducted an anonymised record linkage of SARS-CoV-2 laboratory data from the National Health Laboratory Service's (NHLS) data repository, and clinical data from the South African national hospital surveillance system, DATCOV. DATCOV is an active surveillance system that was established in March 2020 for recording COVID-19 hospitalizations from both public and private healthcare sectors in South Africa (7). Data from both data sources were collected between 01 April 2020–31 Aug 2022 from Gauteng Province (GP), and the only data included in the analyses were for cases whose outcome status was recorded as either “COVID-related death” or “discharged alive”. CDW data were accessed on 02/11/2022 while DATCOV data were accessed on 20/03/2023. The study period was also divided into four pandemic waves as follows: Asp614Gly/wave1 (7 June - 22 Aug 2020); beta/wave2 (15 Nov 2020–6 Feb 2021); delta/wave3 (9 May–18 Sept 2021) and omicron/wave4 (21 Nov 2021–22 Jan 2022).

Clinical data of hospitalized laboratory-confirmed COVID-19 cases from GP with a positive SARS-CoV-2 real-time RT-PCR test or a positive SARS-CoV-2 antigen test were obtained from DATCOV (7). Infection based on a positive SARS-CoV-2 antigen test was subsequently confirmed by molecular testing during hospitalization. A hospital admission was defined according to the definition given by the DATCOV surveillance system, that is, a confirmed hospital stay of ≥1 day regardless of the reason for admission or age (7). All hospitalized SARS-CoV-2 cases from GP reported in the DATCOV surveillance system during the study period, irrespective of age, gender or reason for hospitalization was included in the study. Data recorded in DATCOV included demographic (age, race, sex), exposure, presence of a comorbidity (including diabetes, hypertension, chronic kidney disease, chronic cardiac disease, chronic pulmonary disease or asthma, cancer, tuberculosis or HIV) (8), disease complications, treatment and outcome information (7).

Commercial rRT-PCR assays that were used for SARS-CoV-2 testing during the study period, as well as their respective SARS-CoV-2 specific gene targets were: Roche (ORF gene), GeneXpert (N2 gene) and Abbott (RdRp gene). A laboratory-confirmed SARS-CoV-2 infection was defined as a positive result as per the NHLS virology laboratories' standard operating procedure for SARS-CoV-2 rRT-PCR testing. Only SARS-CoV-2 laboratory results from the NHLS CDW linked to a hospital admission were included in the study. In the case of patients with multiple positive tests during the entire study period, only the Ct value result of the first test conducted immediately before or at hospital admission was included, with all other results belonging to that particular case being removed from the data that would be used in the analyses. SARS-CoV-2 Quality Assurance and SARS-CoV-2 community screening results were excluded. Ct-values were also stratified into three groups: low (Ct < 20); medium (20 ≤ Ct ≤ 30) and high (Ct > 30). These cut-off values were chosen based on cell cultures transfection and viral antigen expression data previously described (6).

Categorical data were expressed as frequencies and percentages, while numerical data were reported as means with their 95% confidence intervals, and results were considered as statistically significant if *p* was ≤0.05.

Following univariable logistic regression, multivariable logistic regression analysis was used to assess the association between Ct-values and COVID-19-related in-hospital mortality by adjusting for the following predictor variables that have been established as COVID-19 risk factors in the literature: age, sex and the presence of a comorbidity. Results with *p* values ≤0.05 were considered as statistically significant, and Stata statistical software version 15 was used for data analysis (StataCorp® College Station, Texas, USA).

This study's ethical approval was obtained from the Faculty of Health Sciences Research Ethics Committee of the University of the Witwatersrand (Ethics reference number M2010108), while institutional clearance was obtained from the NHLS Academic Affairs, Research and Quality Assurance. Consent from patients whose data were used in the analysis was waived by the ethics committee because only anonymised data were obtained from the two data sources.

## Results

A total of 1205 cases met our study criteria: 836 (69.4%) during wave1; 122 (10.1%) during wave2; 21 (1.7%) during wave3; 11 (0.9%) during wave4. The mean age (±SD) of the cases was 49 years (±18), while 662 (54.9%) of the patients were female. Regarding the cases' clinical outcome, 909 patients were discharged alive, while 296 (24.6%) died, with the distribution of deaths according to pandemic wave being: 241 (81.4%) during wave1; 27 (9.1%) during wave2; 6 (2%) during wave3; 2 (0.7%) during wave4 (*p* < 0.001). The cases' median (IQR) length of stay was 6 days (2–11). The majority of samples were tested using the Roche testing platform [1,033 (85.7%)], while 169 (14%) samples were tested using the GeneXpert assay and 3 (0.3%) using the Abbott assay. The median (IQR) Ct values according to testing platform were: Roche 26 (22–30); GeneXpert 38 (36–40); Abbott 21 (16–24).

## Demographic characteristics and Ct-values

Table 1 shows demographic characteristics of the cases. Some COVID-19 cases were recorded in the CDW database as SARS-CoV-2 positive based on positive Ct-values of non-specific targets according to the NHLS' diagnostic algorithm. In such cases the Ct value of the specific targets would have been recorded as “NULL”, and such cases were removed from the data for the rest of the analysis, the results of which are shown in Table 1. Individuals <20 years had the highest mean Ct-value compared to older cases (*p* = 0.02), while cases that died had a lower mean Ct-value compared to cases who were discharged alive (*p* < 0.001).

**Table 1:** Mean Ct values of hospitalized COVID-19 cases according to demographic characteristics.

Variable	Total *N* (1,205)	*N*^a^ (1,141)	Mean Ct-value	95% CI	*p*
Age	** **	** **	** **	** **	**0**.**02**
<20 years	58	54	30.31	27.97–32.66	
20–39 years	321	301	27.55	26.81–28.29	
40–59 years	442	424	27.45	26.85–28.04	
60–79 years	330	310	27.11	26.40–27.82	
≥80	53	52	26.88	25.15–28.62	
Sex	** **	** **	** **	** **	0.56
Female	662	628	27.38	26.89–27.87	
Male	532	503	27.61	27.00–28.21	
Presence of morbidity					0.56
No	851	799	27.57	27.12–28.01	
Yes	354	342	27.32	26.60–28.03	
Outcome					**<0**.**001**
Died	296	284	25.35	24.68–26.01	
Discharged alive	909	857	28.20	27.76–28.64	

^a^ Number with SARS-CoV-2 specific gene targets with Ct-values not recorded as NULL.

Bold values are statistically significant.

Table 2 shows results from univariable and multivariable logistic regression analyses.

**Table 2:** Univariate and multivariate analyses of factors associated with in-hospital mortality amongst hospitalized COVID-19 cases, gauteng province, 01 April 2020–31 Aug 2022^a^.

Variable	Alive		Died			Univariate		Multivariate	
	*n*	%	*n*	%	*p*	OR	95% CI	OR	95% CI
Age					<0.001				
<20	51	94.44	3	5.56	** **	1 (ref)			
20–39	273	90.70	28	9.30	** **	1.74	0.51–5.95	1.44	0.42–4.98
40–59	353	83.25	71	16.75	** **	3.42	1.04–11.26	2.75	0.83–9.16
60–79	152	49.03	158	50.97	** **	17.67	5.40–57.83	**14**.**70**	**4.44**–**48.66**
>80	28	53.85	24	46.15	** **	14.57	4.03–52.71	**11**.**67**	**3.16**–**43.06**
Sex					0.02				
Female	488	77.71	140	22.29	** **	1 (ref)			
Male	360	71.57	143	28.43	** **	1.38	1.06–1.81	**1**.**44**	**1.06**–**1.95**
Presence of comorbidity					<0.001				
No	626	78.35	173	21.65	** **	1 (ref)			
Yes	231	67.54	111	32.46	** **	1.74	1.31–2.30	1.25	0.91–1.73
Ct-values					<0.001				
High (>30)	363	84.22	68	15.78		1 (ref)			
Medium (20–30)	408	70.10	174	29.90		2.28	1.66–3.11	**2**.**25**	**1.60**–**3.18**
Low (<20)	86	67.19	42	32.81		2.61	1.66**–**4.09	**3**.**18**	**1.92**–**5.27**

^a^ Only cases with non-NULL Ct values and known sex.

Bold values are statistically significant.

After adjusting for sex, age and the presence of a comorbidity, factors found to be associated with COVID-19 related mortality were age groups 60–79 years [adjusted odds ratio (aOR): 14.70, 95% CI: 4.44–48.66] and ≥80 years (aOR: 11.67, 95% CI: 3.16–43.06) compared to cases <20 years; being male (aOR: 1.44, 95% CI: 1.06–1.95); as well as Ct values 20–30 (aOR 2.25; 95% CI: 1.60–3.18) and Ct values <20 (aOR 2.25; 95% CI: 1.60–3.18) compared to Ct values >30.

## Discussion

Although some available data have shown that adverse COVID-19 outcomes are associated with lower Ct values (3, 9–13), there is currently no consensus regarding the impact of SARS-CoV-2 Ct values on disease course, prognosis or infectivity, with some studies showing no association between SARS-CoV-2 Ct values and disease severity or mortality (14–16). Also, the use of single positive Ct values in determining the disease stage or prognosis have been cautioned, with multiple, or specifically, serial Ct values suggested to have better clinical utility instead (17). Equally important is the fact that Ct values of different assays are not interchangeable, owing to the inherent assay-specific differences (14). Our study showed that, compared to cases with high Ct values >30, the odds of COVID19-related mortality increased in cases with Ct values ≤30, particularly in cases with Ct values <20.

There is conflicting data regarding the association between SARS-CoV-2 Ct values and age. While there is data showing that older COVID-19 patients had lower Ct values, and therefore higher viral loads by inference, compared to younger cases (18–20), such findings could not be confirmed in other studies (16, 21). Our study showed that the median Ct-values decreased with age, inferring that older patients had higher viral loads compared to those who were younger (*p* = 0.02).

Although the risk for severe clinical outcomes and mortality is higher amongst males compared to females following SARS-CoV-2 infection (22), our study findings did not show a significant difference between the mean Ct values amongst men and women (*p* = 0.55), similar to what has been described in other studies (16, 18, 21, 23). This finding may suggest that, apart from biological sex-based differences between men and women, gender-related determinants of health are likely to contribute to COVID-19 related mortality (22).

Our study showed that there is a relationship between COVID19-related mortality and SARS-CoV-2 PCR gene target Ct values <30, with the relationship being the strongest when Ct values were <20. Although lower Ct values are general understood to infer higher viral loads, an inconclusive relationship between COVID-19 viral loads and disease severity has been described (24). One study demonstrated that the association between disease severity and Ct values depends on COVID-19 cases’ overall burden of comorbidities, with disease severity found to be significantly associated with lower Ct values only in cases with a high comorbidity burden (25). Due to a high burden of comorbidities amongst older patients, this would also explain the likelihood of lower Ct values being described amongst older patients. As mentioned earlier, we found that the median Ct values decreased (inferring higher viral loads) as age increased, and this was also in keeping with the odds of COVID19-related in-hospital mortality being higher amongst cases ≥60 years.

The strength of our study was the use of two large and comprehensive datasets, that provided clinical information as well as the outcome status of all of the cases. The study, however, had a few limitations as well. This study used secondary laboratory data and it was therefore not possible to account for any missing information. Also, any pre-, intra- or post-analytical issues that may have had an impact on the results would have affected the Ct vales, and therefore the study's calculated estimates. Our objective was to determine an association, and not causation. The model that we fitted was also neither predictive nor explanatory, and the purpose of including the established COVID-19 risk factors was to adjust for confounding and not for prediction.

## Conclusion

In our study population, we found that lower Ct values were associated with higher odds of COVID19-related death. However, due to inherent assay differences resulting in Ct values not being interchangeable across different testing platforms, the use of Ct values in informing decisions about disease severity and prognosis is cautioned.

## Data Availability

The data analyzed in this study is subject to the following licenses/restrictions: Only anonymized data were provided to the investigators for analysis. Requests to access these datasets should be directed to National Institute of Communicable Diseases; lucilleb@nicd.ac.za.
